# Impact of translation availability on the patient voice in clinical trials: commentary

**DOI:** 10.1186/s41687-026-01156-4

**Published:** 2026-07-21

**Authors:** Dagmara Kuliś, Sonya Eremenco, Lindsay Hedderly, Diane E. Leek, Ana A. Popielnicki, Emily Potz

**Affiliations:** 1 European Organisation for Research and Treatment of Cancer, Brussels, Belgium; 2 Critical Path Institute, Tucson, USA; 3 RWS, Maidenhead, UK; 4 Eli Lilly (USA), Indianapolis, USA; 5 GlaxoSmithKline (USA), Philadelphia, USA; 6 Suvoda, Conshohocken, USA

**Keywords:** Linguistic validation, Clinical outcome assessment, COA, Translation

## Abstract

For clinical outcome assessments (COAs) to be used in global clinical trials, they must be developed in the native language of participants through the process of linguistic validation (LV). This commentary discusses why ensuring linguistic coverage might be difficult for sponsors and what consequences a lack of translated COAs might have on study data. Access to translations might be challenging due to issues with the licensing of available translations, incorporating translation development processes into study timelines, or not foreseeing language needs of each study during the planning phase. In consequence, poor quality translations might be used, or certain countries or regions might not be included at all, leading to missing data and limitations to the results of the study. To prevent these negative consequences to study data, COAs should be included in the study planning as early as possible, giving all stakeholders adequate time to fulfill any licensing requirements as well as to properly develop measures in other languages to ensure conceptual equivalence and data comparability to support pooling of data across countries. By improving communication and expectations across stakeholders involved in the licensing and translation of COAs for clinical trials, translations will be more widely available for the participants who need them, increasing the presence of the patient voice in clinical trials.

## Background

Patient-focused drug development (PFDD), also known as patient-centered drug development, “marks a shift away from the traditional drug development model of discovering, developing, and marketing medications to satisfy regulatory requirements towards a focus on putting the patient at the center.” [1] Clinical outcome assessments (COAs) are used in clinical trials to assess clinical benefit, defined as “a positive effect on how an individual feels, functions, or survives.” [2] Our commentary intends to address the impact of translation availability, or lack thereof, on the ability to capture the patient voice via COAs. To collect information from patients (“participants” in this context) in multinational clinical trials, translated COAs are essential in providing participants with measures in their native language, ensuring appropriate comprehension of the concepts addressed in the items. This, in turn, generates robust data reflecting the participant’s voice and helps show potential clinical benefit of treatment.

Our aim is to highlight the importance of developing linguistically validated [3] translations according to industry standards to ensure the participant’s voice is captured within clinical trials. We will do this by analyzing the reasons why linguistically validated translations may not be available for a given clinical trial, the consequences, and considerations to be made going forward.

Sponsors might encounter problems with the availability of all language versions of COAs needed for their study for a number of reasons.

### Licensing requirements

While there are a few resources available to assist with finding the copyright holder of a selected COA, such as databases of COAs including the copyright holder’s contact details or publications allowing tracing of the COA’s development, in general, the due diligence task of locating a copyright holder to determine license requirements can be laborious and time consuming, with no comprehensive, global reference available. Timelines needed to reply to queries and process legal documents might also be lengthy, especially when legal discussions are required, and are unpredictable between copyright holders. What is more, copyright holders might place restrictions on the development of unavailable language versions of a COA (for example, requiring an entire item bank to be translated to ensure consistency, considerably delaying the availability of the subset of items needed for a trial as a result). To complicate matters, these restrictions and requirements might not be openly communicated. Further, copyright holders may require the involvement of a third-party provider (such as an exclusive Language Service Provider or a preferred electronic COA (eCOA) vendor) throughout the licensing and translation development process, who may have their own timelines and processes, adding to the complexity of the task.

### Time constraints in linguistic validation

With its multiple steps as described in the industry standard [3], the linguistic validation (LV) process can require more time than is available before the study is scheduled to begin in each country. Sponsors should be aware that LV typically requires cognitive interviews to ensure that the translated COA is conceptually equivalent and culturally appropriate for the target population. This can lead to country start-up delays as this important step is complex and time-consuming. Additionally, if a country is added at a later stage and a new translation is needed, it might be difficult to expedite the process sufficiently to incorporate it into the life cycle of a study. Finally, the lack of clarity over ethics committee submission requirements, whether paper or deliverables based on other modes of data collection, can further impact the time needed to produce the finalized translations.

### Difficulty foreseeing language needs

Sponsors face a number of challenges when preparing country participation lists for trials. In the case of major, well-established migrant groups in specific countries, it is easier to foresee the need for translations being available at sites or in the eCOA system. However, in recent years, due to unforeseen events such as military conflicts resulting in refugee populations or mass migration, clinical teams might need to accommodate speakers of languages in countries where these languages are not officially spoken. This is also the case for individuals who are migrating to places that offer treatments unavailable in their country of origin. Because translations into these languages were not accounted for in trial or project planning, there might be limited or no time for development to satisfy emerging and evolving needs for exigent, unplanned circumstances. While we are not advocating for developing country-specific translations for recently displaced immigrant populations, previously published recommendations [4] have attempted to address this issue. Finally, given that the process of LV is both time- and cost-consuming, the investment of resources for the needs of a few patients might be deemed an inefficient allocation of limited budgets.

### Consequences of lack of translations

There are several negative consequences to a situation where translations are not available for a trial. In some cases, participants may be recruited to take part in other aspects of the study but cannot contribute COA data if the COA is not available in their language. In such cases, in order to win time, workarounds resulting in poor translations, performed in a sub-optimal way and not including all the necessary steps of the LV process, may be suggested by various parties. When used, such workarounds might lead to a lack of conceptual equivalence, rendering the translation and collected data invalid. More often, a decision is made not to administer COAs in the country or linguistic region for which the translation is not available. As a result, these participants are classified as “missing data” for the COA endpoint of the trial, which might lead to insufficient data to make conclusions about the impact of a treatment on the participants in the study or require sample size increase in other countries to account for the “missing data” – with each bearing their costs in terms of the inability to demonstrate the study meeting the COA-based endpoint, or investing additional financial and time resources to account for the insufficient sample. Such systematically missing data can compromise the integrity of the study by skewing representation toward countries where translations are available, potentially distorting the observed treatment effect and limiting the applicability of findings across global populations. Such results may not be accepted by all regulatory authorities because the data are not representative of all countries participating in the trial, potentially requiring additional data collection, once the translation has been developed – adding time to the approval timelines as well as substantial additional cost. Therefore, the clinical benefit of the treatment could be perceived as not fully evaluated due to the lack of COA data in certain countries, which means a potentially promising treatment may fail not due to lack of efficacy, but because of insufficient data due to the absence of properly translated COAs.

## Conclusion

The absence of linguistically validated translations creates cascading negative consequences that compromise COA data integrity, regulatory acceptance, the inability to run trials in certain countries (bearing negative economic consequences of their own), and ultimately patient access to potentially beneficial treatments. COAs and their translations are still often an afterthought in the clinical trial planning process. Instead, COAs should be considered as part of the overall clinical trial endpoint strategy early in the trial planning process, and sponsors need to be aware of and plan time for licensing of COAs as well as for proper LV in other languages. This will ensure conceptual equivalence and data comparability to support pooling of data across countries. Instrument developers need to find a compromise between being scientifically robust about specific requirements that delay the availability of translations and being pragmatic when it comes to making translations available. By improving communication and expectations across all parties involved in the licensing and translation of COAs for clinical trials, translations will be more widely available for the participants who need them, increasing the presence of the patient voice in clinical trials.

## Data Availability

No datasets were generated or analysed during the current study.

## Abbreviations

COA: Clinical outcome assessment
eCOA: Electronic clinical outcome assessment
LV: Linguistic validation
PFDD: Patient-focused drug development
